# Atlanta Society of Medicine

**Published:** 1897-04

**Authors:** 


### SOCIETY REPORTS.

### ATLANTA SOCIETY OF MEDICINE.

ATLANTA, GA., March 2, 1897.

Regular meeting of the Atlanta Society of Medicine.

The Society was called to order by the President, Dr. G. H. Noble.

The regular order of the meeting was a paper by Dr. J. S. Todd, who spoke on the "Relations of Boards of Health to the Prevention of Tuberculosis."

Dr. Todd said: Boards of Health are resisted, we know, by the people everywhere, but they are important factors in preventing diseases. Cholera is now almost entirely confined to its Asiatic home by the boards of health. We do not fear the bubonic plague on account of boards of health. We can remember when fights were made against quarantining against yellow fever; but all now recognize the value of such measures, for we know that it has been practically driven from the United States by the action of the boards of health. I think, therefore, that we should sustain and uphold these gentlemen who strive for the public good as the members of no other profession do.

By the investigations of Koch we now have a knowledge of the cause of tuberculosis. This disease is widespread, and perhaps more so where the people are crowded together. It is stated by the most noted pathologist of Vienna that from sixty to seventy per cent. of all who die show evidences of tuberculosis. It is not so active here, but I am informed that between thirteen and fourteen per cent. of all diseases in Atlanta are traceable to tuberculosis. It is said that in the United States one-fourth of all septic diseases are traceable to it. We know that the disease is caused by the tubercle bacillus. This germ conforms to the laws of Koch: it is found in every case, in every lesion, can be cultivated in pure culture, and is accounted for by its distribution. That being the case, the people need education in regard to it. Tuberculosis is the most wide-spread of diseases, and it is growing more and more prevalent. They say the increase has been fifty to seventy per cent. in the last few years in the colored race. It is a preventable disease, and therefore I think every case should be reported. It is estimated that about two billions of the germs are expectorated by every consumptive in twenty-four hours. We know that the bright sunlight will destroy them, but that they thrive in shaded places, and especially damp places. Therefore every workshop becomes a source of infection.

I am glad to say that it is not a hereditary disease. The old ideas in regard to this have been entirely overthrown. Very few are born with the disease, and these are so few that the exception proves the rule. Those formerly supposed to inherit the disease were simply surrounded by an atmosphere of tuberculosis and were infected by contact. A child of tuberculous parents, if put where it cannot inhale the infection, will not have the disease. We say that stone-cutters and those who engage in similar work are the most susceptible to the disease. This is due to the irritation of the lungs by the dust, etc., making a suitable soil for the germ. Every street-car that we go into is very apt to be the source of infection. I think the sleeping-car is one of the worst places. The consumptive expectorates on the curtains, and any one having an acute cold or an ordinary case of catarrh runs the risk of being infected. The people need instruction on this subject. Many doctors will resist it, just as they resisted vaccination; but now in New York we have a practical illustration of the benefits to be derived. The issuing of circulars to the people would cost almost nothing, and if the people just knew how they can be infected we could diminish the death-rate. If a person knew he was a walking death-trap to the community, he would gladly take any necessary precautions. In New York twelve years ago they began this system, and it is reported that the disease has diminished thirty per cent. there. They have now passed a resolution compelling the doctors to report every case.

I read not very long since an article showing how very infectious the air is in which a patient has tuberculosis. Five guineapigs were inoculated with the dust from the room of a tuberculous patient, and three of them died of tuberculosis. From another room which had been reported to the board of health and which had been fumigated, five other guinea-pigs were inoculated and none of them were affected.

Now, are the doctors to ignore all these facts and not attempt any prophylactic measures against such diseases? Not by any means; it is our duty to mitigate suffering and endeavor to make life more enjoyable. We are slumbering under the impression that consumption is not a disease of southern latitudes; but we can have it here as well as in any other country if we do not take precautions. We know that in Aiken, S. C., Florida, Colorado, and other places which had been supposed to be exempt from the dissease, it is now becoming prevalent. There is a little place in France where it was once noted that the inhabitants were free from infectious diseases, and it was supposed that it was impossible for them to exist there. It was recommended as a resort for consumptives, and they flocked there; the inhabitants waited on them, washed their clothes, etc., and now it is said that the original inhabitants, within the course of fifty years, have been almost exterminated, and that it is the most tubercular spot in all France.

Tuberculosis is an infectious disease, but it can be prevented; and I make this talk, gentlemen, simply to solicit views from the profession as to what they think about it. Consumption is so prevalent that we do not recognize it as we should. In a post mortem recently at the Grady Hospital I was surprised to find tubercular lesions when there had been no evidence to excite suspicion of their existence.

*Dr. Kendrick:* From what knowledge I have of this I indorse everything that Dr. Todd has said in regard to the contagiousness of tuberculosis. It is a specific infectious disease as far as our knowledge goes; as specific and infectious as any disease of which we have knowledge. I indorse every word he has said in regard to the pathology. In 1883 Koch made his investigations and established the facts in regard to this disease, and these investigations have hardly been added to or shaken, but remain as he left them twelve or fifteen years ago, and with Dr. Todd, I believe that the physicians should fall into line, and that the people should be instructed so as to guard against the expectorations, which, although not the only source of infection, are yet the main one. If we could control all the expectorations we could certainly diminish the extent of the disease in a number of years. All expectorations should be made into spit-cups or cloths and not allowed to dry, for, as a rule, the danger is not in the moist state, but after the sputum becomes dry. The bacillus becomes attached to particles of dust and these are inhaled, and the bacillus is found in the lung. You may take the dust from the room in which a patient has died of the disease and expectorated about the room and inject this dust into guinea-pigs and they will die in a reasonable length of time with the disease. I remember that Osler states that cotton was placed in the nostrils of nurses and this was afterwards examined and in some sixty per cent. the bacilli were found. As Dr. Todd stated, I do not believe that the disease is transmitted from the mother to the child, but that we inherit the soil and if we come in contact with the bacilli we have the destruction of the lung tissue and finally the death of the patient. By controlling the sputum we may not obliterate the disease, but I believe that it could be lessened about seventy-five per cent. I have stated before in this society that it is shown that from forty to fifty per cent. of people forty-five years of age we will find with tubercular nodules. These may be quiescent and become walled in or calcified and the patient will go on never knowing that tubercular lesions existed and die in old age. As Dr. Todd states, it is a wide-spread disease, and one out of every seven dies of some tubercular condition, either of the lungs, bones, or other structures of the body. Small-pox and other diseases have been almost completely eradicated, and we could also circumscribe tuberculosis and lessen it to a great extent in a few years. There is no doubt that every one of us every day inhale the bacilli in the dust in the city. As Dr. Todd stated, the street-cars and sleeping-cars are a great source of infection. We are not infected because we do not have the soil.

*Dr. Hagan:* I am always in thorough sympathy with progressive medicine. I have nothing especial to mention, but will simply relate an incident of a recent trip. There were three consumptives in the same sleeper as myself and they coughed and expectorated to such an extent as to keep me awake. The man next to me coughed and expectorated 117 times and 36 of these struck the curtains and several times he missed the cuspidor. I thought then that there was no greater hotbed for tuberculosis than the sleeping-car. We might take precautions at home, but this source of infection would remain.

*Dr. Jones:* I do not think any of us would question Dr. Todd's position in regard to tuberculosis. I understand his position is to back up the board of health in all infectious diseases. I think we may make a mistake in attempting to put too much on the board of health and lose sight of individual responsibility. I recall one case of where the wife contracted tuberculosis from her husband and died of the disease. Now if this husband had been instructed in time he would have gladly taken all precautions, and this is where our individual responsibility comes in. I am in full accord with Dr. Todd's views and think we ought to sustain the board of health ; at the same time we ought not to lose sight of our responsibility. When a patient is notified through a public body in regard to these matters, they are going to object, as they will think their private matters are being made public, and therefore we can do a great deal ourselves.

*Dr. C. D. Hurt:* I call attention to the fact that some fifteen or sixteen years ago in Vienna the profession, or a large number of the intelligent members of the profession, were disposed to take the position that there was never a case of pneumonia that did not have tuberculosis as its basic origin. I think what Dr. Todd has mentioned is nearer the truth. We have in this climate, in these Southern States, a peculiar condition in the way of preventing this disease, and a peculiar opportunity for developing it; that is, in the negro race, we have a people who have a tendency to a lack of cleanliness, and who often live at the top of the ladder one day and at the bottom to-morrow; they will have plenty to-day and to-morrow it will be the reverse, thus living a very irregular life. These conditions have a tendency to develop more tuberculosis in the negro race, in this section of the country, than in the whites. I do not know the percentage, but it is greater in the negro. This refers to those white people living here, for we have a number coming here as consumptives. As far as my experience goes the disease is on the rapid increase. We ride in the same cars with the negro and are thrown more or less with them, are therefore subjected more or less to the poison expectorated by them, and the increase in the whites is due in a great degree to the negro. One of the greatest acts of benevolence of the medical profession would be the development of the plan to control the expectorations of the disease, and this I think can be done by educating the laity.

Speaking of the contagiousness of the disease, I knew a gentleman who was in perfect health, and he married a lady with tuberculosis, and he contracted the disease and died three years before she did. All of her family, parents, brothers and sisters, died with it. It was said then that they inherited it, but we now know this to be an error. However, I do not belive that the question of heredity should be wiped out. I see no reason why it could not be inherited, but I think the percentage of inherited cases is small compared to those due to contact.

*Dr. Todd:* I thank the doctors for the kind reception my talk has met with. Undoubtedly objections will be raised. I may have expressed myself too strongly in regard to having the board of health report immediately all cases of infectious diseases. In regard to the physician warning the patients, they will not believe; but to have it sanctioned by the board of health would have greater effect. I remember when I came to Atlanta and told people that the clear, cool water which they were drinking out of the wells was unhealthy they would not believe it. This had to be taken up by the board of health; and if they had not taken it in hand the people would yet be drinking water obtained within the fire limits. We need first a campaign of education, and we should warn the people about the disease. For a long time we did not know positively as to its cause. A great many people do not yet believe that such a thing as bacilli exist. Dr. Jones is right; we ought to talk more about it.

There are other sources of infection besides dust and the respiratory tract. Wounds can be infected, milk can convey infection, and in other ways, but the expectoration is the main one. The board of health is to gradually bring up these things, sustained by the moral support of the profession. You will find that if the board of health starts a campaign it will be opposed.

*Dr. Jones:* I would like to introduce the following:

"*Resolved,* That the Atlanta Society of Medicine is in hearty accord with the Board of Health in its efforts to suppress contagious diseases in the city of Atlanta, and that the members of the Society pledge their earnest support to such measures as the board may adopt, and that they would hail with delight the dissemination of literature calculated to enlighten the public, especially in reference to the communicability and prevention of tuberculosis."

Unanimously adopted.

*Dr. Kendrick:* I want to ask whether the Woodbridge treatment for typhoid fever is practiced in this community to any extent. If we were to practice anything along that line to cut short typhoid fever, then we must set aside the pathology. Now, the bacilli of Eberth are rarely found in the contents of the intestinal canal until the second week, but they are found in the walls of the intestines and get into the lymph follicles, or Peyer's patches, and their presence there excites the inflammation, and they do not get into the excretions until the second week. Therefore any treatment that would cut short the disease would set aside the pathology.

*Dr. Richardson:* I do not know to what extent this treatment is practiced. I know only one man that has tried it. I have read his book, and I think it is very inconsistent. I do not agree with it. I do not think it is possible to disinfect by any means the alimentary canal, and this is the basis of his treatment. His claims are wild, absurd, and irrational, and remind somewhat of the quack. There are a great number of cases of so-called typhoid fever in this climate that run their course in twelve to fourteen days. Woodbridge would call these cases of typhoid fever that were aborted. The treatment is not practiced to any great extent in Atlanta.

*Dr. Duncan:* I have had the temerity to use the Woodbridge treatment and to give the medicine every fifteen minutes. Just prior to beginning the use of the treatment I had some patients to die. I then had two cases of typhoid fever about closing the first week. I put both on the Woodbridge treatment, tablet number one every fifteen minutes while awake. One was a married man and the other single. The single man's sister had died just a few days before. That young man's bowels on the next day moved about twelve times. He looked much better, and gradually improved until within eight days he was free from fever. He had a temperature of 103 and 104. He was up after the eighth day, and began to eat regular meals and had a relapse. The second attack was about as severe as the first, and lasted about eight days. The other man had a severe attack of the fever, and he made a continuous gain from day to day until free from fever.

After this I had a number of cases of typhoid fever, and put them on the Woodbridge treatment straight, and I have not lost a single case since I began the treatment, and I have had grave cases. In every case their features improved and they felt more cheerful. You may say what you please about the treatment, but there is something in it. Over here on Houston street I was called to see a case of sickness, and found one patient dead, and in another bed was a patient which I thought would be dead in two days. They had been under the treatment of a young physician, and I told them to send for him. He consented to put this patient on the Woodbridge treatment, and the patient made a good recovery. Three others got sick with the fever, and one of these was a stout man, and I gave him a prescription, and when I saw him two days later found that he had not been able to buy the medicine. I then had him sent to the hospital, where he died in less than two weeks.

Dr. Jones moved that the President appoint a committee of three who shall investigate the arrival of quacks; also practitioners, and examine their credentials. He stated that we had gone to a great deal of trouble to get a Medical Board, but they simply examined applicants for license and did not look up new arrivals. Those practicing illegally should be reported to the board so they can be prosecuted. Motion carried.

---

SCENE in Jewish clothing house: Customer—"Say, Ike, this suit of clothes I bought from you yesterday is full of moths." Ike—"Vell, vat do you expect for six tollars und a quarter? humming berds?"—*Ex.*

